# Supplementary figures and images for: Maniraptoran pelvic musculature highlights evolutionary patterns in theropod locomotion on the line to birds
Source: PeerJ. 2021 Mar 4;9:e10855. doi: 10.7717/peerj.10855 (PMC7937347; doi:10.7717/peerj.10855)

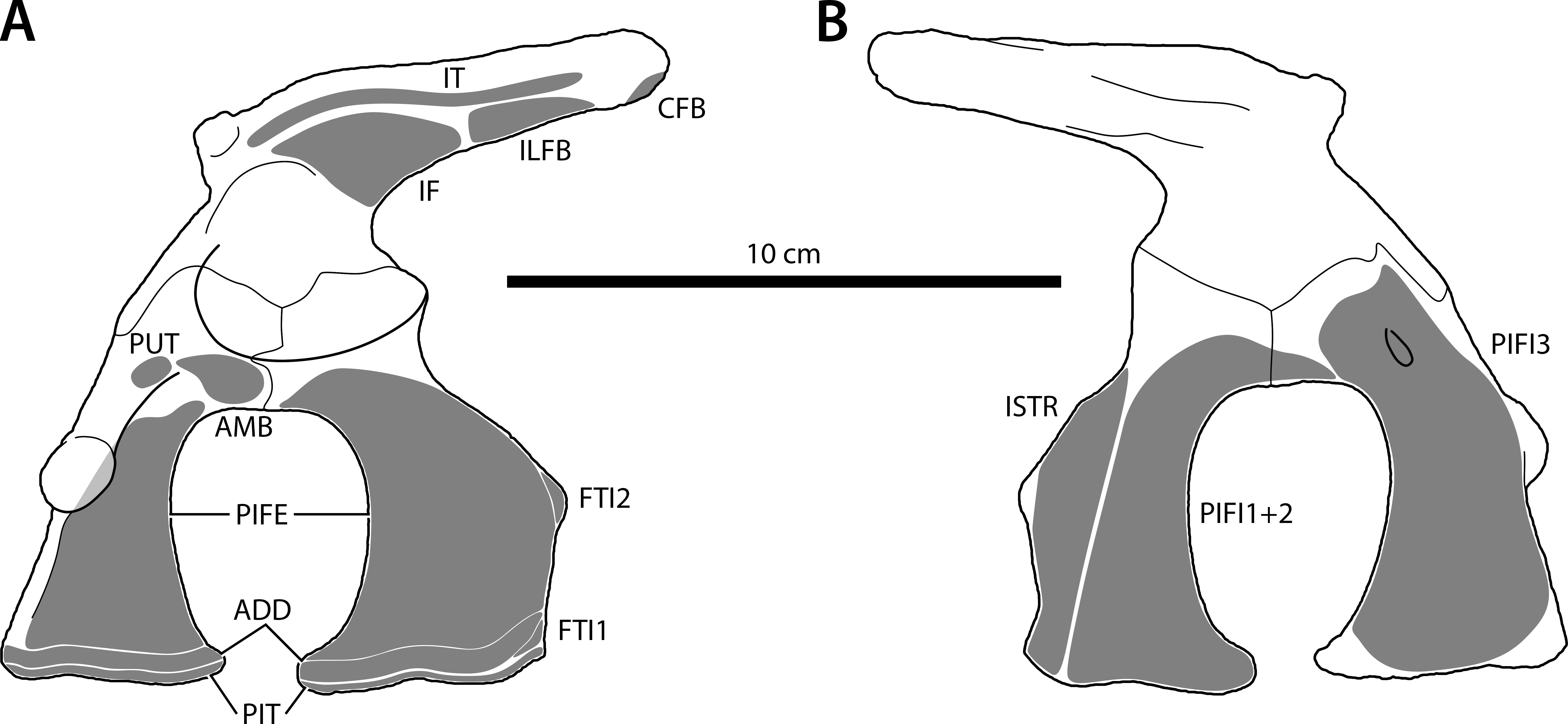

Supplement: Supplemental Information 1 — (A) Pelvis in left lateral view. (B) Pelvis in medial view. See Table 2 in article for muscle abbreviations. [file peerj-09-10855-s001.png]

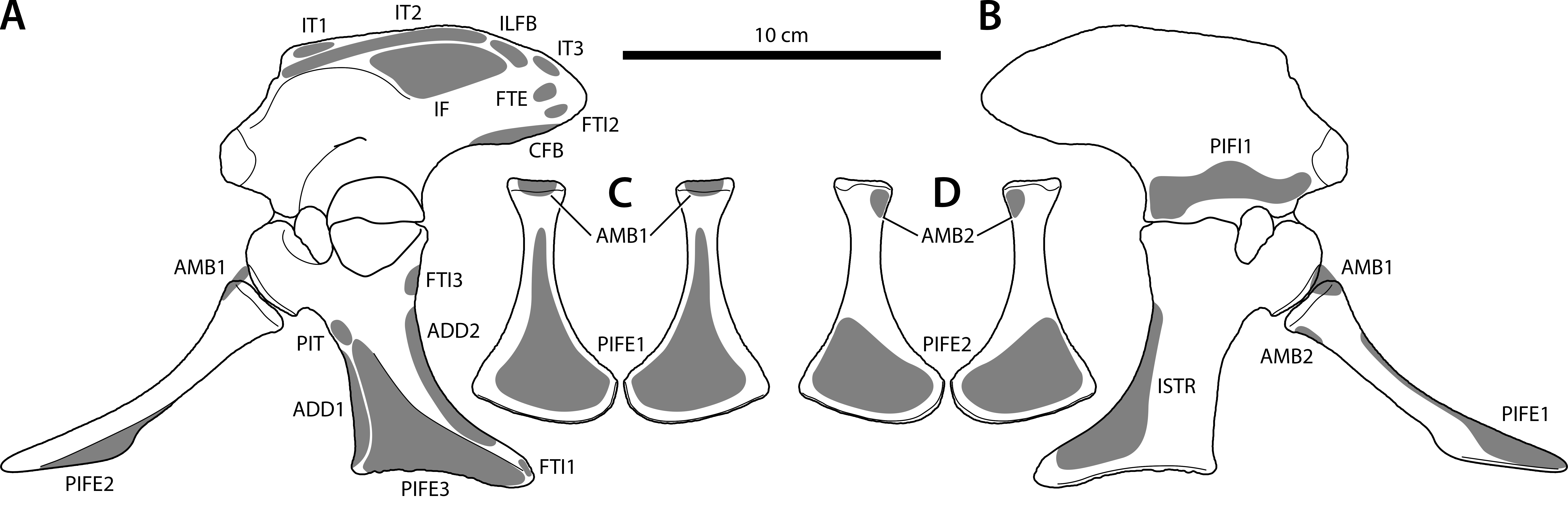

Supplement: Supplemental Information 2 — (A) Pelvis in left lateral view. (B) Pelvis in medial view. (C) Pubes in dorsal view. (D) Pubes in ventral view. See Table 2 in article for muscle abbreviations. [file peerj-09-10855-s002.png]

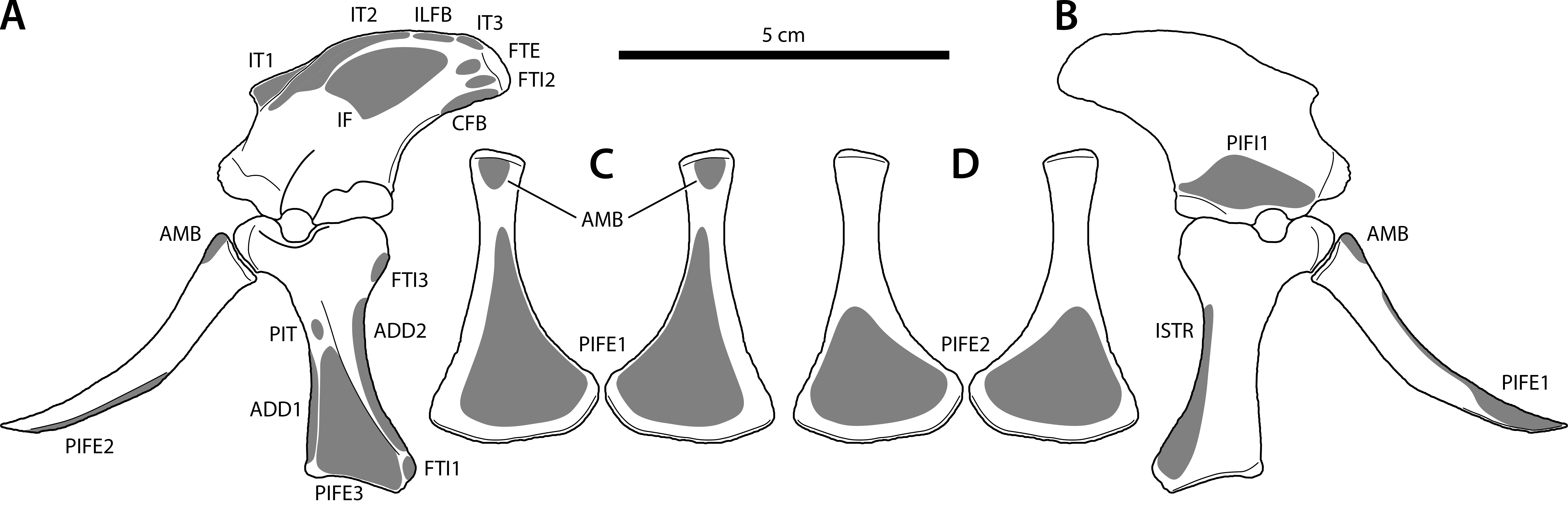

Supplement: Supplemental Information 3 — (A) Pelvis in left lateral view. (B) Pelvis in medial view. (C) Pubes in dorsal view. (D) Pubes in ventral view. See Table 2 in article for muscle abbreviations. [file peerj-09-10855-s003.png]

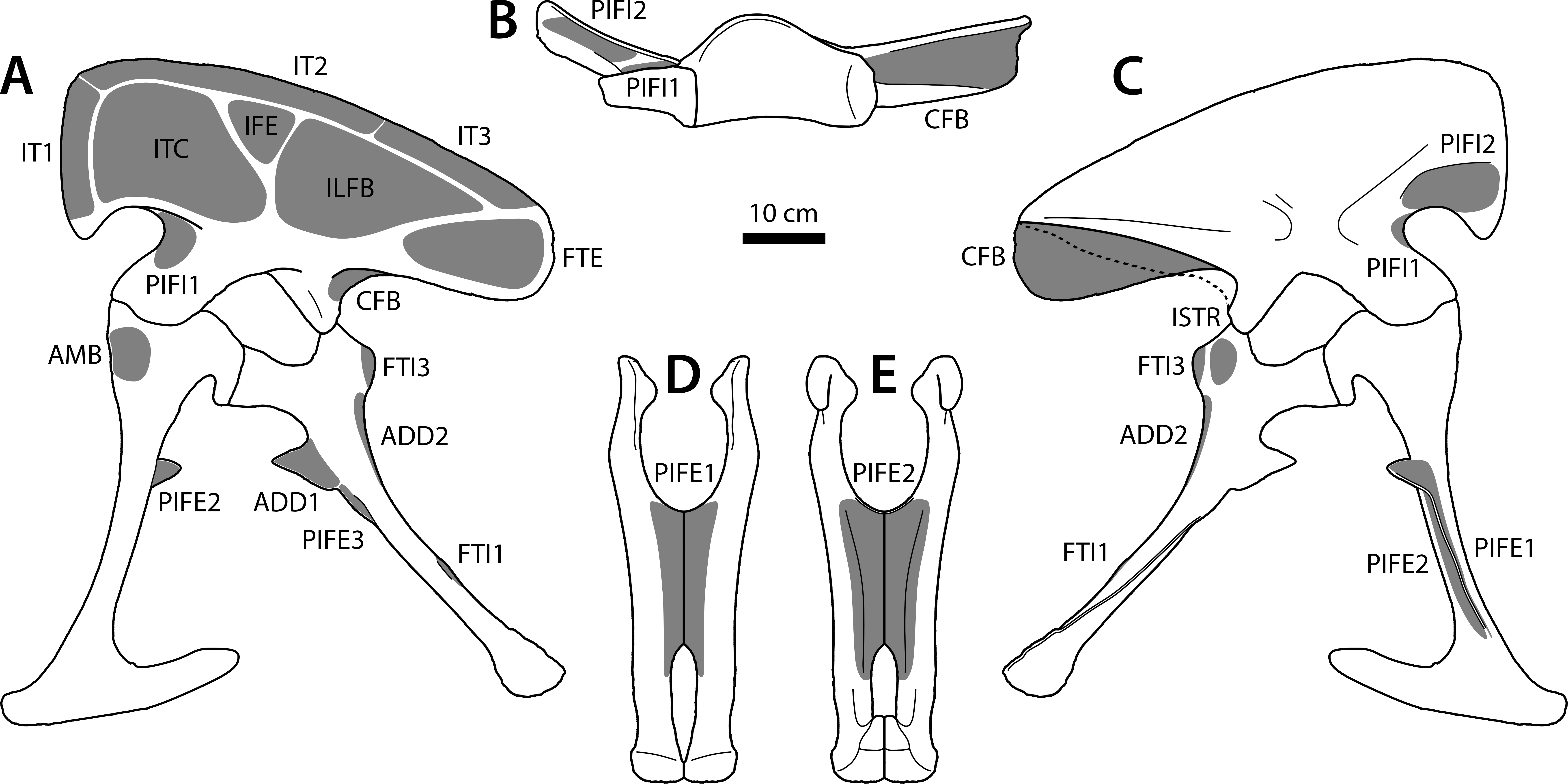

Supplement: Supplemental Information 4 — (A) Pelvis in left lateral view. (B) Ilium in ventral view. (C) Pelvis in medial view. (D) Pubes in anterior view. (E) Pubes in posterior view. Dashed line in medial view indicates cutaway to show the extent of the brevis fossa. See Table 2 in article for muscle abbreviations. [file peerj-09-10855-s004.png]

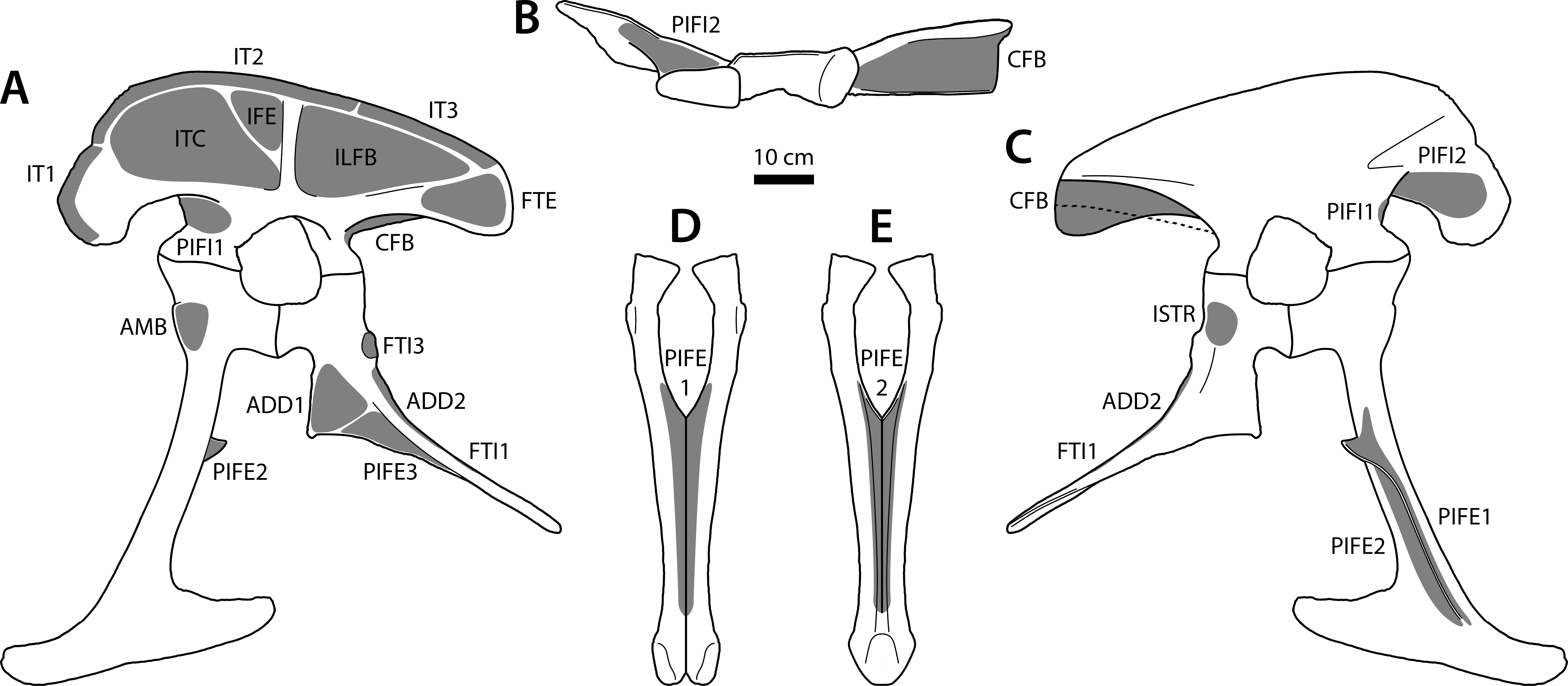

Supplement: Supplemental Information 5 — (A) Pelvis in left lateral view. (B) Ilium in ventral view. (C) Pelvis in medial view. (D) Pubes in anterior view. (E) Pubes in posterior view. Dashed line in medial view indicates cutaway to show the extent of the brevis fossa. See Table 2 in article for muscle abbreviations. [file peerj-09-10855-s005.png]

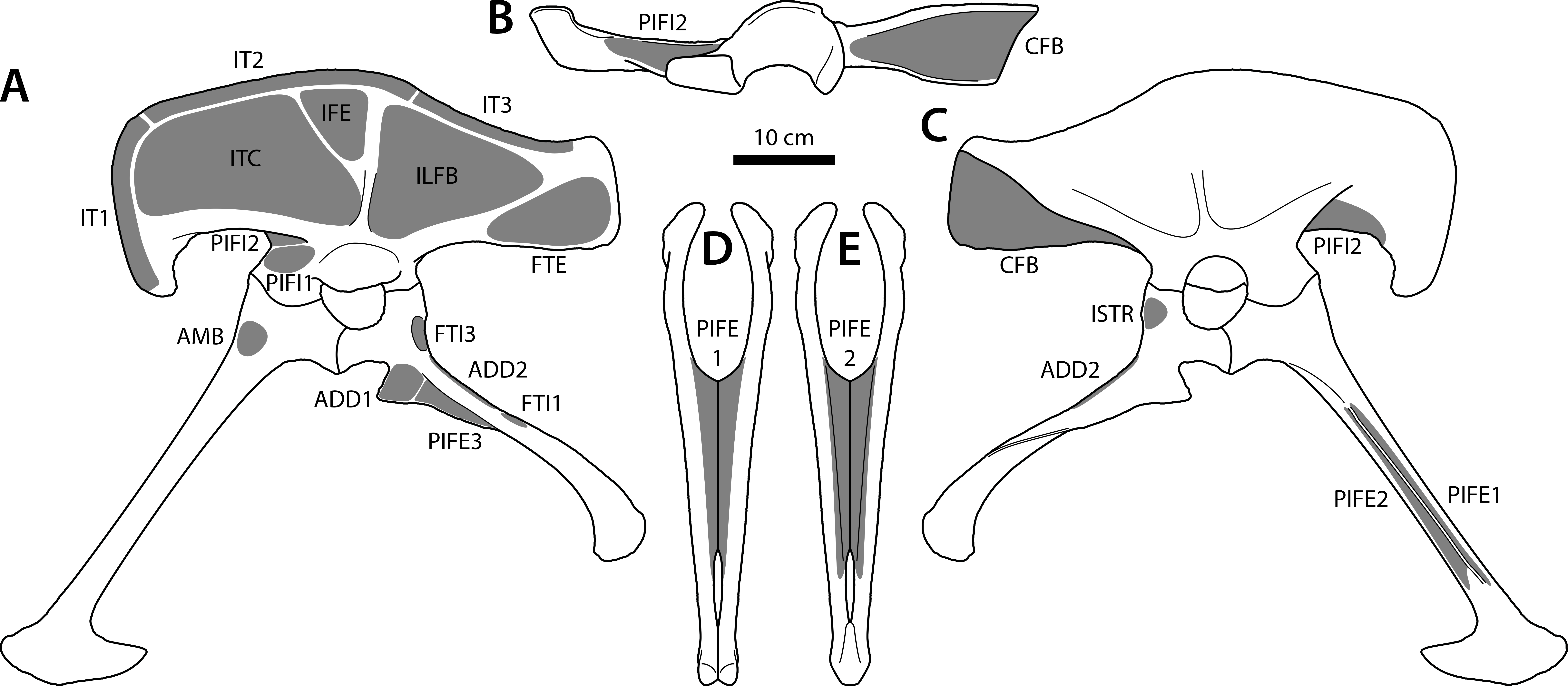

Supplement: Supplemental Information 6 — (A) Pelvis in left lateral view. (B) Ilium in ventral view. (C) Pelvis in medial view. (D) Pubes in anterior view. (E) Pubes in posterior view. See Table 2 in article for muscle abbreviations. [file peerj-09-10855-s006.png]

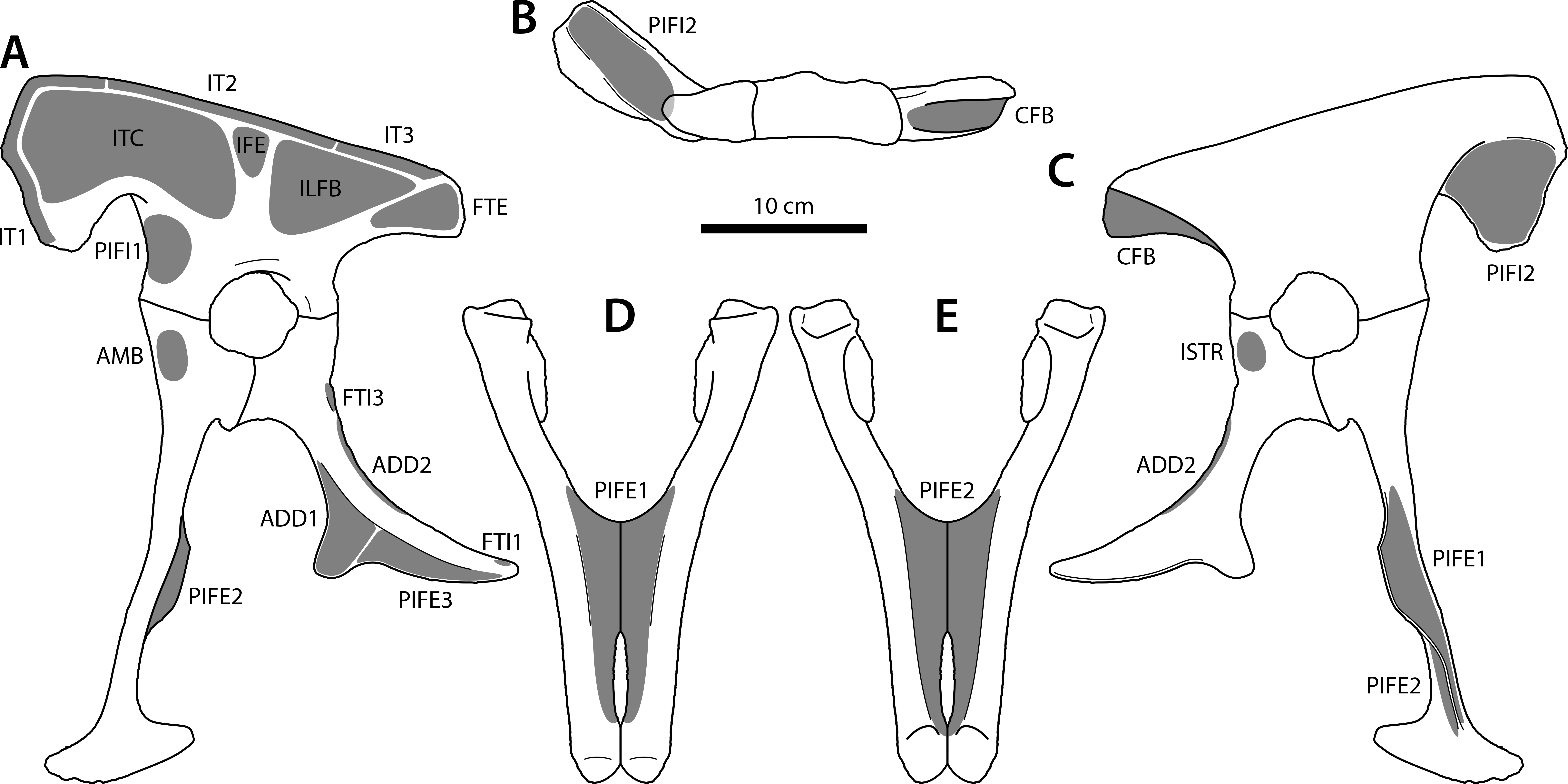

Supplement: Supplemental Information 7 — (A) Pelvis in left lateral view. (B) Ilium in ventral view. (C) Pelvis in medial view. (D) Pubes in anterior view. (E) Pubes in posterior view. See Table 2 in article for muscle abbreviations. [file peerj-09-10855-s007.png]

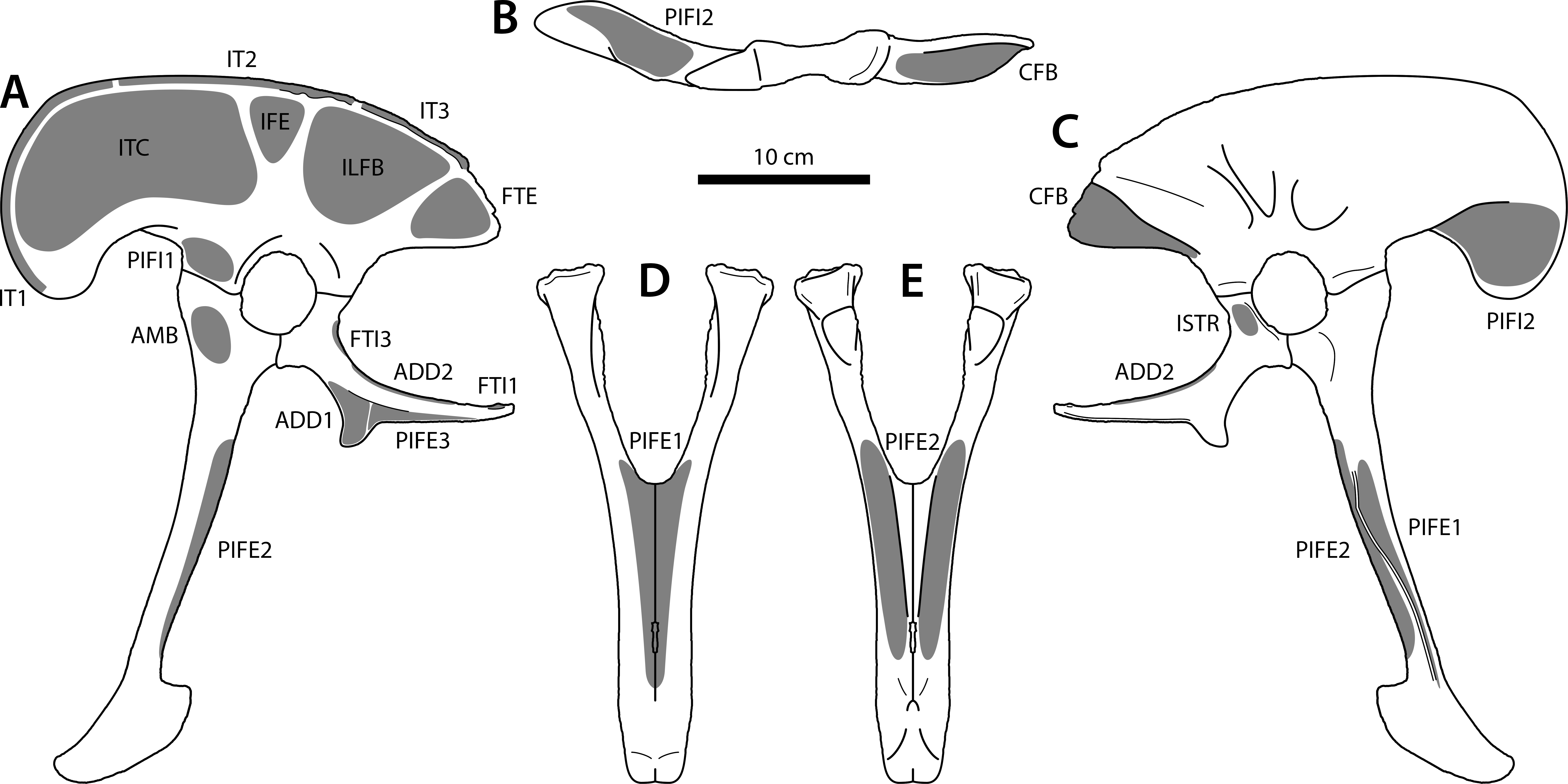

Supplement: Supplemental Information 8 — (A) Pelvis in left lateral view. (B) Ilium in ventral view. (C) Pelvis in medial view. (D) Pubes in anterior view. (E) Pubes in posterior view. See Table 2 in article for muscle abbreviations. [file peerj-09-10855-s008.png]

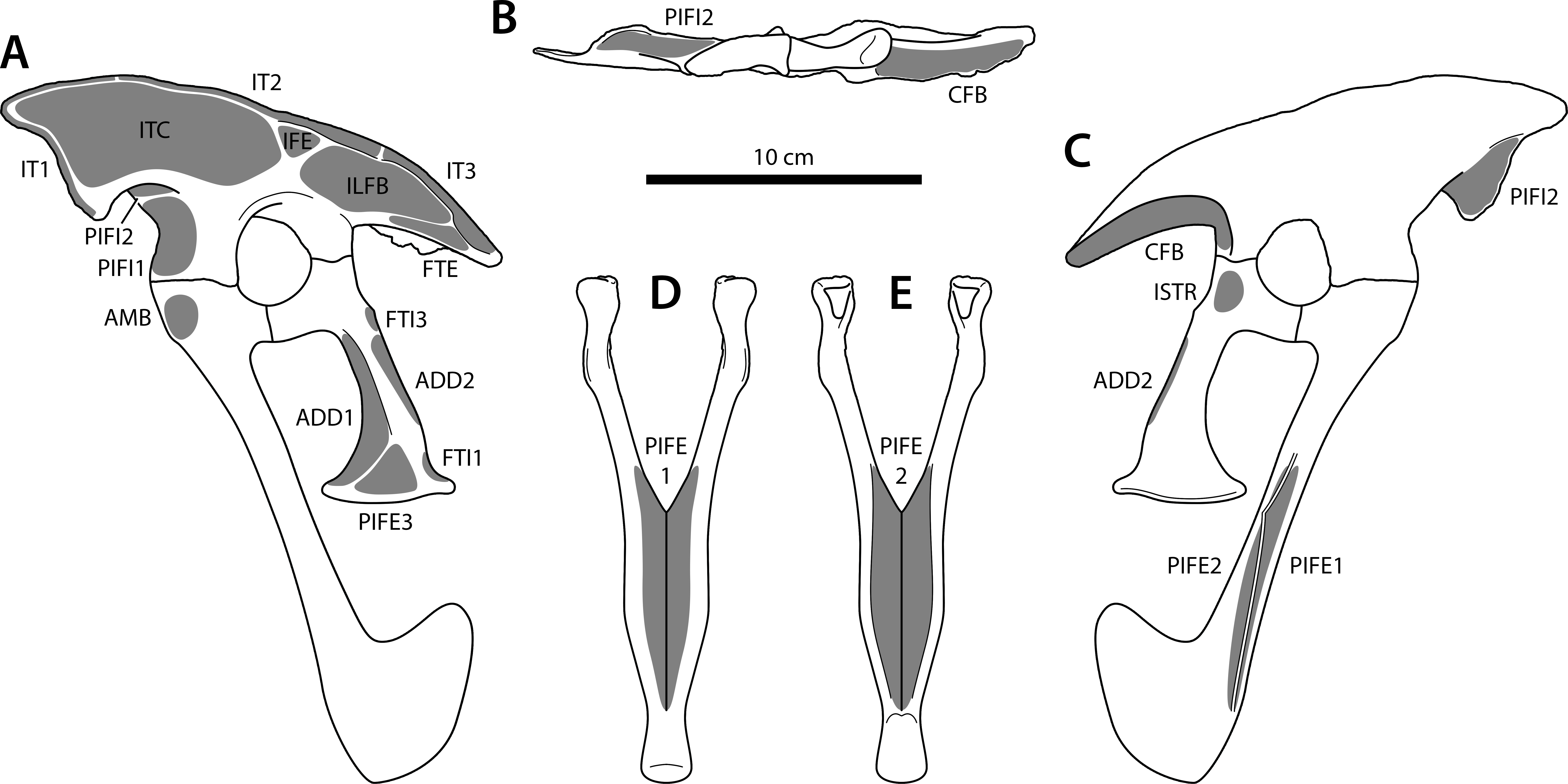

Supplement: Supplemental Information 9 — (A) Pelvis in left lateral view. (B) Ilium in ventral view. (C) Pelvis in medial view. (D) Pubes in anterior view. (E) Pubes in posterior view. See Table 2 in article for muscle abbreviations. [file peerj-09-10855-s009.png]

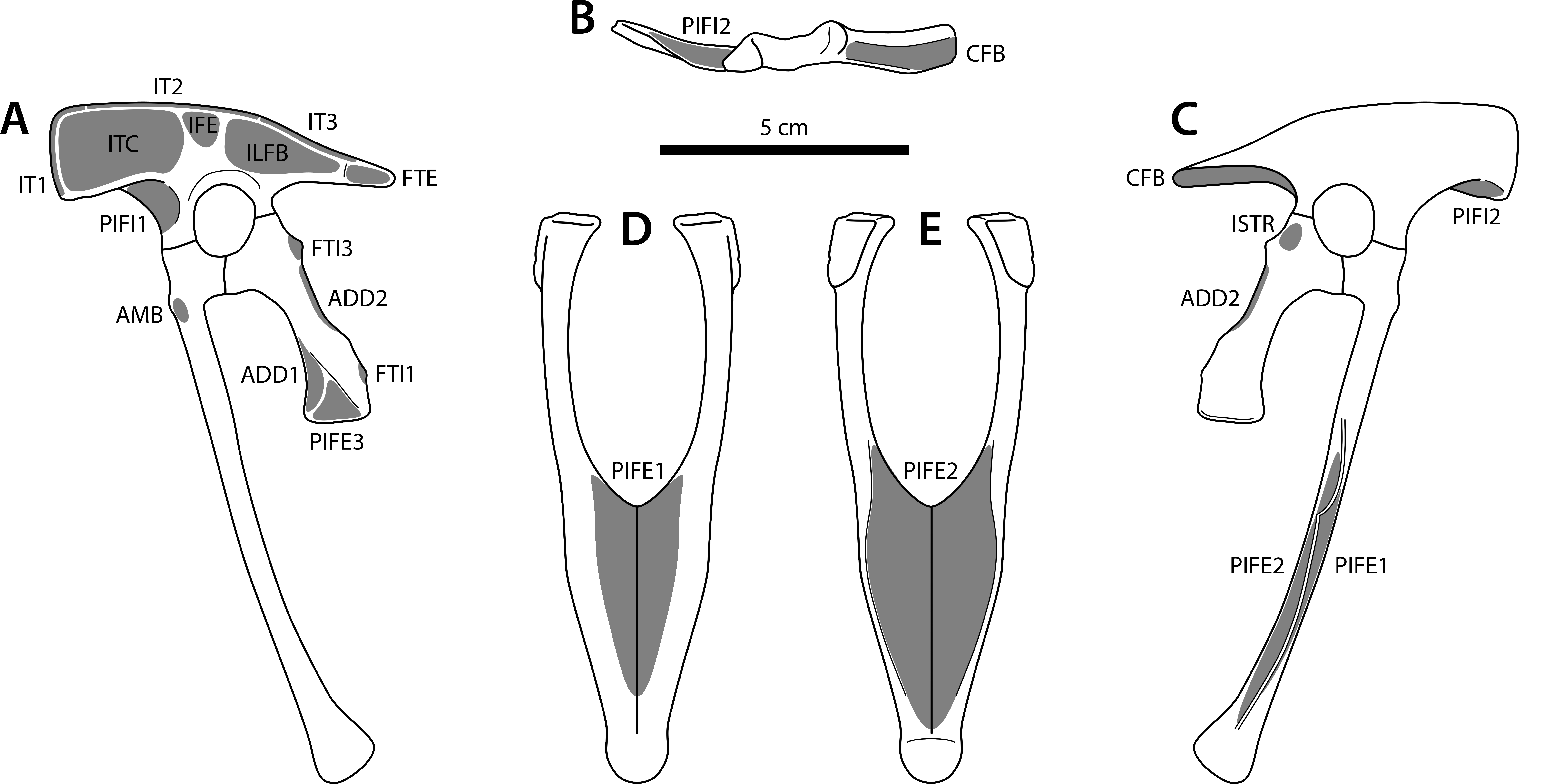

Supplement: Supplemental Information 10 — (A) Pelvis in left lateral view. (B) Ilium in ventral view. (C) Pelvis in medial view. (D) Pubes in anterior view. (E) Pubes in posterior view. See Table 2 in article for muscle abbreviations. [file peerj-09-10855-s010.png]

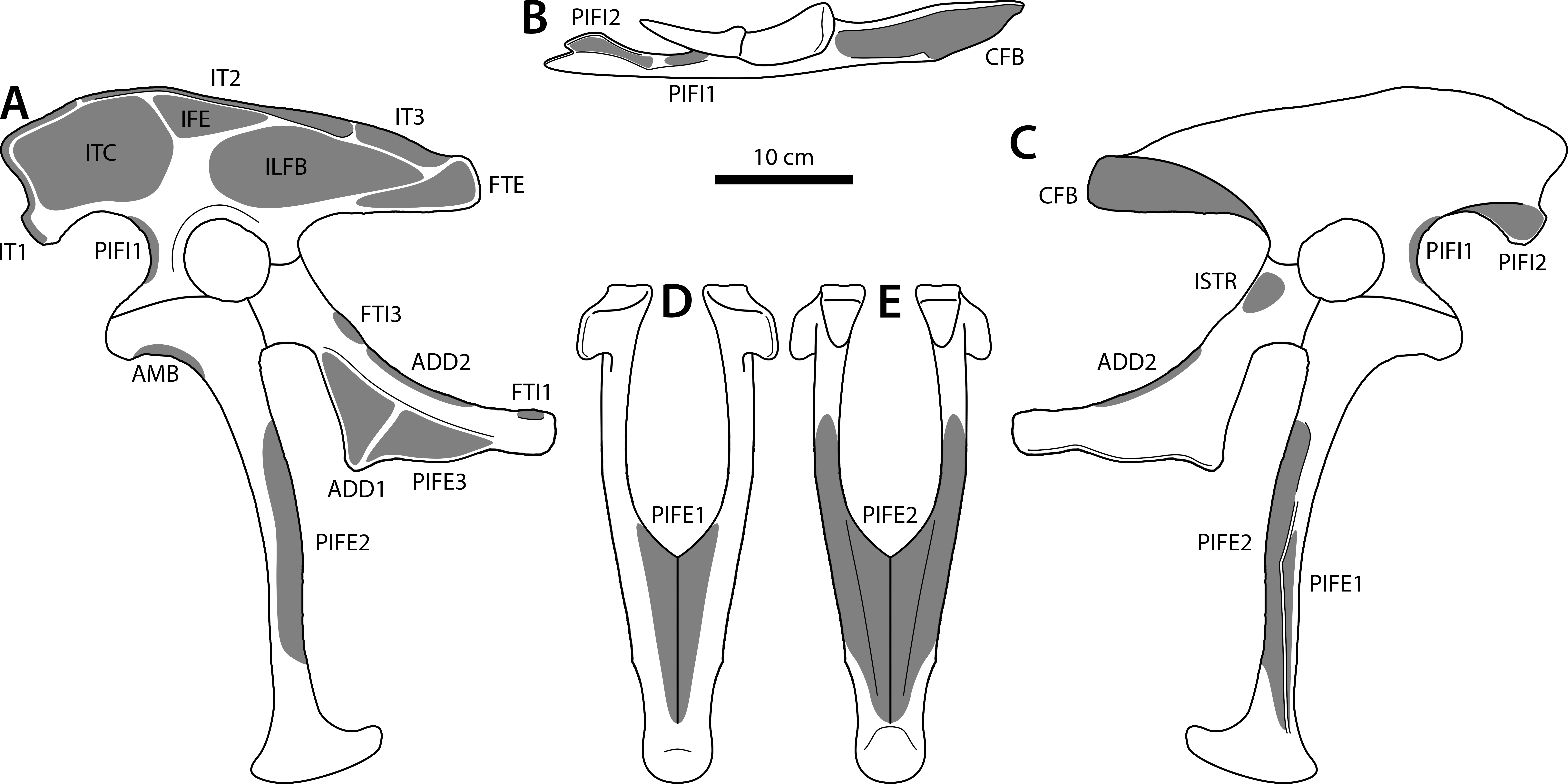

Supplement: Supplemental Information 11 — (A) Pelvis in left lateral view. (B) Ilium in ventral view. (C) Pelvis in medial view. (D) Pubes in anterior view. (E) Pubes in posterior view. See Table 2 in article for muscle abbreviations. [file peerj-09-10855-s011.png]

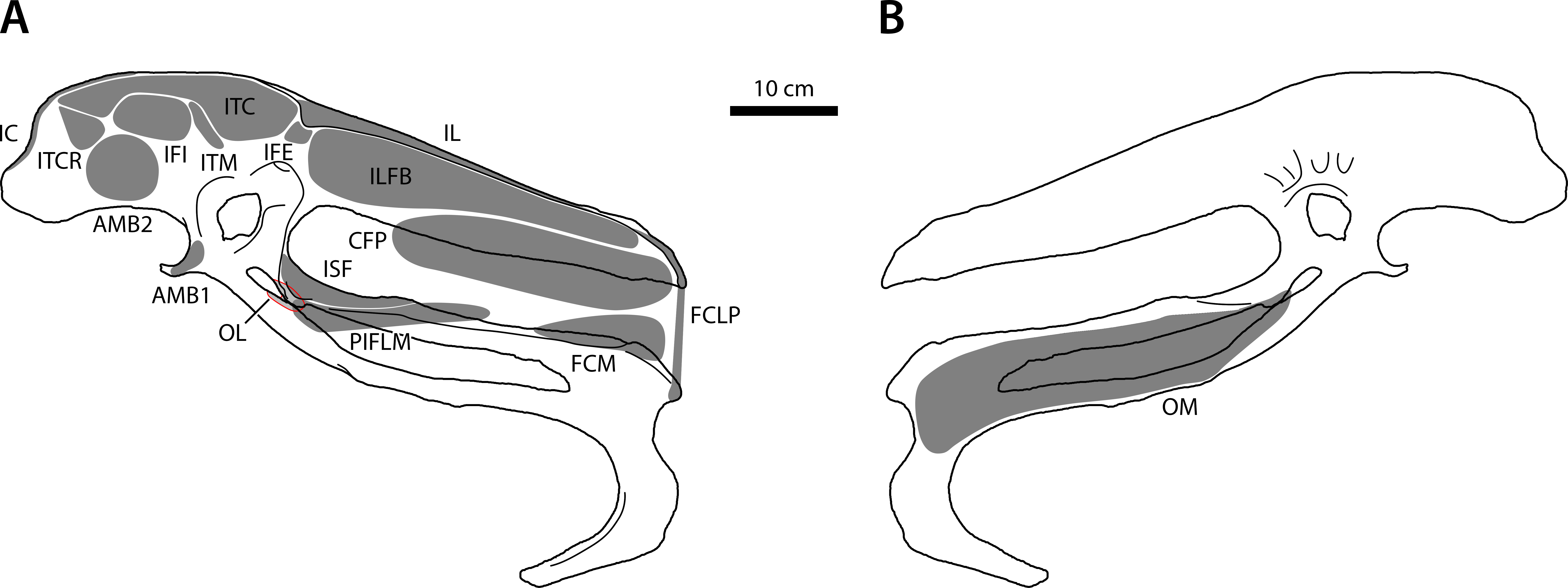

Supplement: Supplemental Information 12 — (A) Pelvis in left lateral view. (B) Pelvis in medial view. See Table 2 in article for muscle abbreviations. [file peerj-09-10855-s012.png]

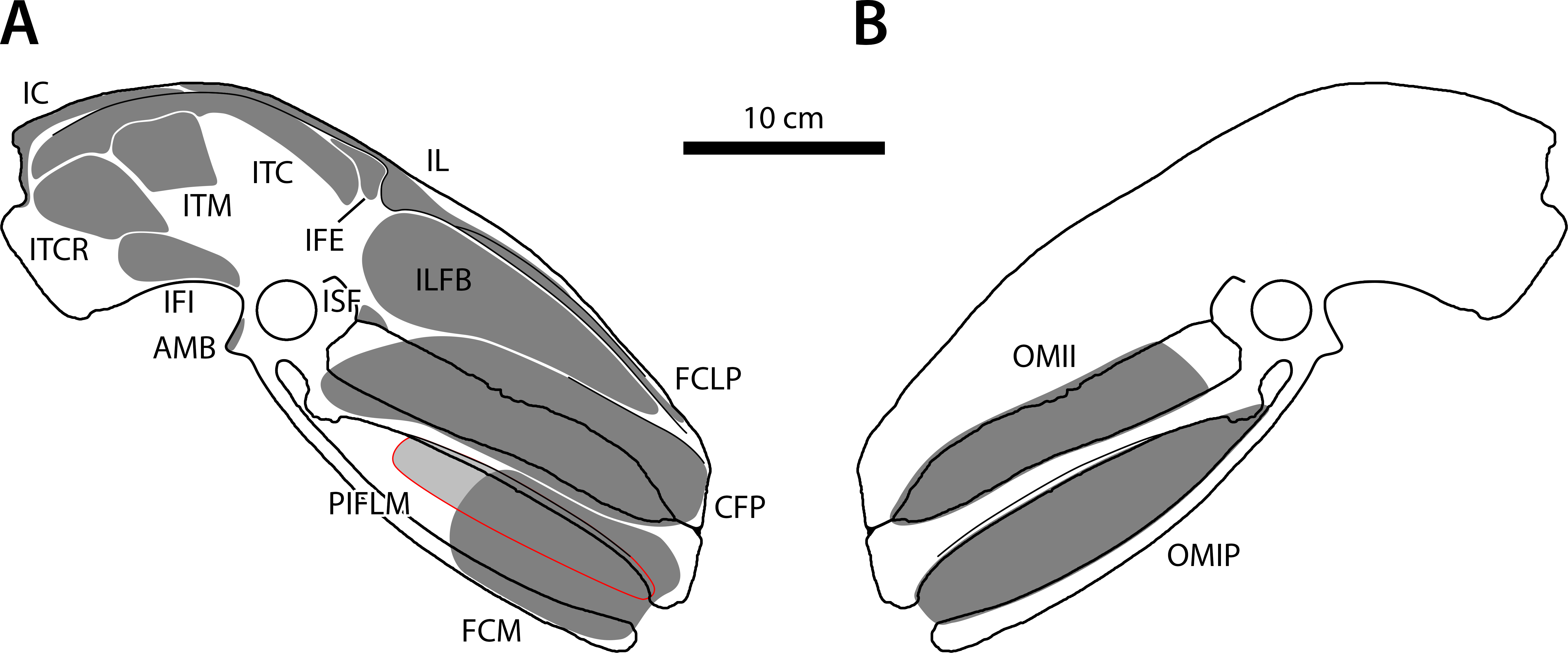

Supplement: Supplemental Information 13 — (A) Pelvis in left lateral view. (B) Pelvis in medial view. See Table 2 in article for muscle abbreviations. [file peerj-09-10855-s013.png]

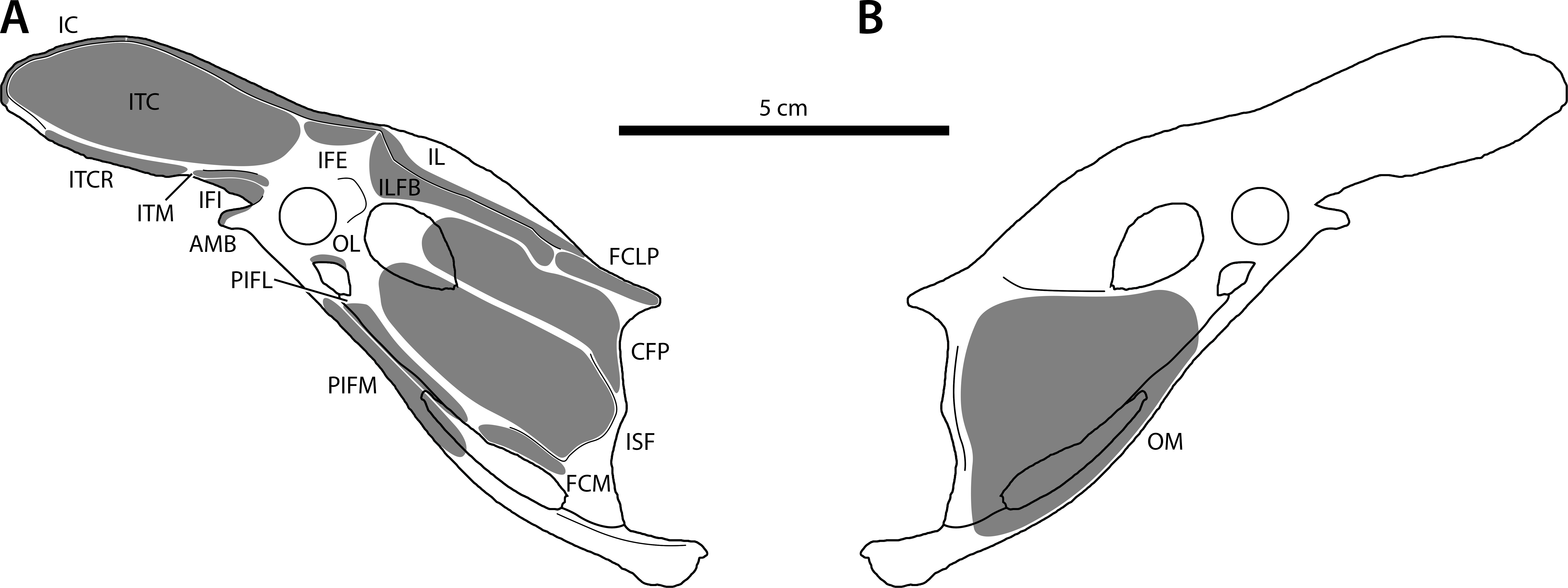

Supplement: Supplemental Information 14 — (A) Pelvis in left lateral view. (B) Pelvis in medial view. See Table 2 in article for muscle abbreviations. [file peerj-09-10855-s014.png]
